# Innovative Predictive Approach towards a Personalized Oxygen Dosing System

**DOI:** 10.3390/s24030764

**Published:** 2024-01-24

**Authors:** Heribert Pascual-Saldaña, Xavi Masip-Bruin, Adrián Asensio, Albert Alonso, Isabel Blanco

**Affiliations:** 1 Advanced Network Architectures Lab (CRAAX), Universitat Politècnica de Catalunya, 08800 Vilanova i la Geltrú, Spain; adrian.asensio@upc.edu; 2 Fundació de Recerca Clínic Barcelona-Institut d’Investigacions Biomèdiques August Pi i Sunyer (IDIBAPS), 08036 Barcelona, Spain; aalonso@recerca.clinic.cat; 3 Department of Pulmonary Medicine, Hospital Clínic, University of Barcelona, 08036 Barcelona, Spain; iblanco2@clinic.cat

**Keywords:** chronic obstructive pulmonary disease COPD, artificial intelligence, machine learning, edge computing, blood oxygen saturation, personalized modeling, edge predictions

## Abstract

Despite the large impact chronic obstructive pulmonary disease (COPD) that has on the population, the implementation of new technologies for diagnosis and treatment remains limited. Current practices in ambulatory oxygen therapy used in COPD rely on fixed doses overlooking the diverse activities which patients engage in. To address this challenge, we propose a software architecture aimed at delivering patient-personalized edge-based artificial intelligence (AI)-assisted models that are built upon data collected from patients’ previous experiences along with an evaluation function. The main objectives reside in proactively administering precise oxygen dosages in real time to the patient (the edge), leveraging individual patient data, previous experiences, and actual activity levels, thereby representing a substantial advancement over conventional oxygen dosing. Through a pilot test using vital sign data from a cohort of five patients, the limitations of a one-size-fits-all approach are demonstrated, thus highlighting the need for personalized treatment strategies. This study underscores the importance of adopting advanced technological approaches for ambulatory oxygen therapy.

## 1. Introduction

Chronic health conditions, such as cardiovascular diseases, cancer, chronic respiratory problems, or diabetes, are the leading causes of death worldwide and significantly impact the quality of life and disability of affected individuals [1]. Focusing on chronic respiratory diseases, chronic obstructive pulmonary disease (COPD) is the major contributor to the global burden, with over 3.23 million deaths recorded in 2019, with 80% of these deaths occurring in low- and middle-income countries [2]. The average prevalence of COPD, as reported by European countries to the World Health Organization (WHO) from 2017 to the present, is between 1 and 2% and is expected to increase significantly by 2050 [3]. For example, it is forecasted that the prevalence of it will reach up to 13.8%, with a total of 4.8 million patients in Spain and up to 13.1% in United Kingdom. These predictions indicate a concerning trend of COPD becoming a major public health issue in the coming decades, highlighting the importance of continued research and development of effective interventions and treatments for this condition.

Long-term oxygen therapy (LTOT) is a common and well accepted treatment for patients with COPD and, in general, for patients with any other respiratory disease [4,5,6] tha strongly impacts patients’ capacity to breath [7,8]. Consequently, the main rationale behind LTOT is to improve this patients’ capacity to breath, thereby reducing breathing difficulties. To this end, patients are demanded to carry an oxygen concentrator, which is responsible for providing patients with the theoretically needed supplementary amount of oxygen. In order to measure these breathing difficulties, clinicians consider blood capillary peripheral oxygen saturation (SpO_2_), which is the indicator used to assess the need for oxygen and conceptually stands for the fraction of oxygen-saturated hemoglobin relative to the total hemoglobin [8]. The supplementary oxygen flow rate prescribed to patients by clinicians is fixed and static and therefore, with a high level of probability, will not always match real patients’ oxygen needs [9]. This static dose is decided by clinicians applying a rule-based approach [8] that is based on the SpO_2_ values obtained from patients running some well-known exercise tests (the most usual being the 6-min walking test, 6MWT). These tests are conducted periodically (e.g., twice per year) by patients in hospitals to adjust or modify their oxygen prescription based on the updated state of the disease [8].

However, the mismatch between the real oxygen needs and the oxygen provided often leads to either hypoxemia during exertion or hyperoxemia when resting. Put briefly, hypoxemia can cause adverse health effects such as dyspnea, muscle fatigue, and decreased exercise tolerance [10]. On the other hand, hyperoxemia, or an excess of oxygen in the blood, can cause health problems such as oxygen toxicity, which can damage the lungs and other organs [11]. Therefore, it would be extremely important to monitor in real time the SpO_2_ levels in patients receiving LTOT and to dynamically adapt the oxygen flow rate to the real needs of patients in order to ensure that they always receive the appropriate amount of oxygen to avoid the problems mentioned above (see [12] for a more detailed analysis).

To address these challenges, the first approach might aim at designing an intelligent oxygen dosing system to dynamically and reactively deliver the appropriate dose of oxygen at the right time. In this paper, we go far beyond this first approach and propose to to consider a reactive approach but rather to consider a proactive one that intends to personalize the treatment for each individual patient based on their specific physical conditions and the physical activity to be undertaken [13].

The reactive approach envisioned above is not new. Indeed, recent hospital tests have employed closed-loop oxygen dosing systems with good results [14]. These systems use control strategies based on proportional–integral–derivative, proportional–integral, or rule-based approaches to regulate the oxygen supply to a predefined set point. The oxygen dose is adjusted as a direct function of SpO_2_, with satisfactory short-term results [15]. However, as expected, changes in oxygen saturation levels are not instantaneous and it takes several seconds for the delivered oxygen dose to affect the SpO_2_ level [16], thus resulting in patients continuing to experience shortness of breath. Additionally, several tests that we have also conducted emphasized some fast and unexpected changes in the observed SpO_2_ curve, which may turn into errors in the closed-loop decision process, thereby strongly affecting patients’ wellbeing. Aligned to our view on the need for a personalized dosing strategy, Kofod et al. in [17] conducted a study that demonstrated the benefits of dynamic oxygen dosing. The authors concluded that “individualized automated oxygen titration decreases dyspnea during walking and increases walking endurance in patients with COPD on LTOT”.

The proactive approach proposed in this paper may efficiently address current limitations in the oxygen dosing system. To this end, by leveraging current tools and techniques available (e.g., edge–cloud continuum and artificial intelligence, AI) the strategy proposed in this paper will take advantage of the data generated by patients to create a personalized oxygen dosing system that will proactively adjust the dose to each patient’s specific situation. The system can predict future changes in SpO_2_ levels based on past experiences and properly adjust the oxygen dose instantaneously to prevent heavy drops or increases in SpO_2_. This adaptive approach can notably revolutionize the management of oxygen therapy for patients with chronic respiratory diseases, significantly improving patient wellbeing and their quality of life while reducing the burden of disease, extending the life of concentrators and their components, and ultimately benefiting the healthcare system. It is also critical to mention that the proposed proactive approach may be deployed on hospital premises but is specifically designed for ambulatory use, thus clearly impacting on real daily patient activity.

The remainder of this manuscript is as follows: Section 2 provides a review of the related work in the subjects directly related to the system. Section 3 provides an overview of the proposed system, an end-to-end solution for personalized modeling, and real-time edge SpO_2_ predictions. AI life cycle-relevant steps are detailed in Section 4, while the methods employed for the hypothesis validation are described in Section 5. The results obtained are presented and discussed in Section 6. Finally, Section 7 highlights the conclusions extracted and also outlines future directions and potential enhancements of the proposed system, thereby contributing to the advancement of personalized medicine in the field of respiratory disorders.

## 2. Related Work

In recent years, predictive models have gained significant attention in the respiratory field, with numerous studies conducted to improve the accuracy of predictions. Early contributions in this field date back to 2013 and were written by H. Elmoaqet et al. in [18], and considerable progress has been made to enhance the predictive capabilities of these systems since then.

H. Elmoaqet et al. worked on predictive modeling for many years by applying mathematical methods even though no significant progress was obtained [19,20]. Their last contribution in the area [21] introduced a k-step predictive model utilizing AI for the first time. The authors proposed a framework for multi-step-ahead predictions of critical levels in physiological signals and developed a new performance metric for validation purposes (e.g., prediction accuracy). Results demonstrated a remarkable improvement compared with standard autoregressive models.

Another recent study (Sam Ghazal et al. [22]) proposed the use of different machine learning (ML) techniques to predict five SpO_2_ levels toward a proactive mechanical ventilator setting adjustment in an intensive care unit (ICU). The authors used an artificial neural network (NN) based on the back-propagation method along with a Bootstrap aggregation of complex decision tree implementation as the classifier. While the average results appeared promising, precision for medium and high severity events, which are the most important ones, was low, thereby highlighting the need for having better data quality and quantity to achieve acceptable results from predictive approaches.

It is also worth mentioning that whatever predictive technology may be applied, the IT infrastructure should be able to properly accommodate the specific set of computation and storage requirements. The fog-to-cloud paradigm [23] (today also referred to as edge-to-cloud or more generically as cloud continuum) was introduced as a baseline technology for predictive systems. Xavi Masip et al. applied this paradigm to the health field and more particularly to the LTOT arena by utilizing patients’ contexts, historical data, and biological signals to proactively predict their oxygen dose needs in an optimal infrastructure utilization paradigm, which was centered on computing the results through edge devices [24]. This approach alleviates network load and distributes computing resources in a more efficient manner, demonstrating the feasibility of running AI models in edge devices using currently available technologies.

More recently, H. Pascual et al. [25] provided an initial analysis aimed at predicting blood oxygen saturation by utilizing patients’ vital signs. To this end, a small size test with four patients was considered. The main objective was to assess the applicability of the same proposed AI architecture, which was trained with the same algorithm across diverse individuals, and analyze the obtained results. After analyzing the collected data, the authors concluded that, as expected for the sake of optimality, the same AI model architecture is not universally applicable to all patients. This assessment contributes to the rationale which this paper is supported through, thus driving the set of tests performed in this work and concluding on the need (see Section 6) for considering distinct inputs to build accurate SpO_2_ prediction AI models.

In the commercial arena, some products in the oxygen flow rate regulation field may be found, such as the O_2_ Flow Regulator by Dima Italia™ [26] and Sanso Via™ by Sanso Health [27]. However, although these solutions focus on regulating patients’ O_2_ flows, both of them are completely reactive (closed-loop) and only available on hospital premises. Another device on the market is iGo_2_ [28], an oxygen concentrator designed to slightly adjust the oxygen dose to the patient’s activity through a rule-based system monitoring the respiratory rate. To the best of our knowledge, there is limited research available specifically addressing the challenges of personalized modeling and edge-computed SpO_2_ predictions in the respiratory disorders area. The preliminary tests using the AI model input patterns presented in this paper undoubtedly contribute to filling in this gap by both proposing a complete personalized AI modeling solution and conducting a second small-scale assessment through the AI-input data shape test to obtain clear evidence of the envisioned benefits.

## 3. Early Stage Software Architecture

### 3.1. Architectural Layers

**Figure 1:**
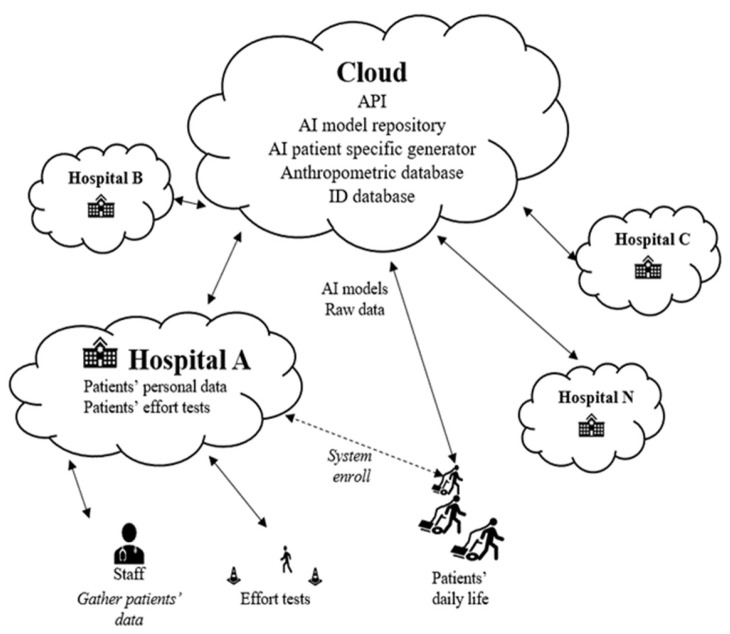
Architecture’s overview.

Following up the initial conceptual software architecture proposed in [25] (see Figure 1), this paper goes deep into the functionalities envisioned for each architectural component. To summarize, the main objective of the proposed architecture is to build an easy mechanism for generating a personalized AI model for patients with LTOT by leveraging the data collected from the individual patient at the edge of the system. This model is responsible for proactively predicting the estimated oxygen titration for each individual patient in the near future according to the patient’s status and the activity the patient is performing. To achieve this goal, many tasks are distributed across various locations in the cloud continuum to optimize and properly allocate access to the necessary computational and network capabilities. This computational task distribution is carried out while also considering data security, privacy concerns, and resource availability, assuming the fact that medical data are especially sensitive information.

**Figure 2:**
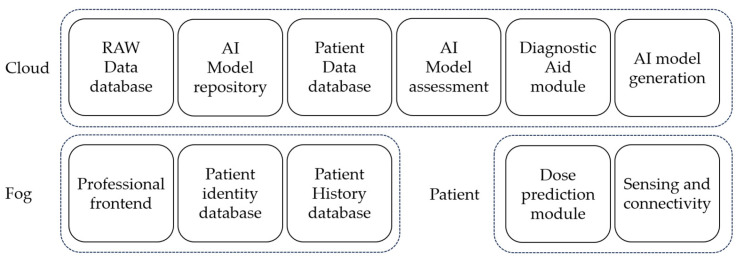
System’s architecture block diagram in the three computational layers.

Figure 2 illustrates the block diagram corresponding to each component of the system, considering the cloud continuum paradigm as the baseline computational technology. Details on each layer and the depicted modules are demonstrated next in this section. Within the Closing Section, the key objectives for the final architectural design are also defined.

### 3.2. Cloud Layer

Several functional modules are located in the cloud layer, considering the fact that cloud encompasses the required computing power and data storage needed for the data analysis and AI training. The main functionalities for the included modules are described next.

**RAW data database:** The RAW data database stores patient data anonymously. Indeed, since the overarching goal of this architecture is to generate models and share information among different hospitals and patients, data privacy becomes a must. Patient-generated data are sent to the cloud (or edge, to be determined) while using the system and stored in this module using an anonymous UUID for future utilization in the AI model generation, AI model assessment, and/or diagnostic aid modules.

**AI model repository:** The AI module repository is a collection of stored AI models for patients with many objectives. It may either maintain a historical record for a patient or provide a model from another patient’s history to a new patient with shortness of data to create a personalized model. Despite not being the optimal solution, a suboptimal model identified by clustering several patients’ disease features in the diagnostic support module would be preferable to a closed-loop system in oxygen dosing.

**Patient data database:** The patient database contains anthropometric data and other functional test results such as, for example, data collected from an exercise test (e.g., A6MWT [29]). These data are needed for the diagnostic aid module for clustering and analyzing patients’ states and disease stages and for the AI model repository to track patients’ models as it contains the system’s patient unique ID, as detailed in Section 3.2.

**AI model assessment:** The AI model assessment module analyzes the accuracy of deployed models. Using the AI model repository and the RAW data database, it must distinguish when a model becomes obsolete for a patient due to many factors like using a very recent model that needs adjustment with fresh personalized data or changes in the patient’s condition. If the module detects an inaccurate model, it requests the AI generation module to generate a new one for the patient.

**Diagnostic aid:** The diagnostic aid module allows professionals to monitor patient progress, make comparisons with similar patients, and gain insights from any part of the system that contribute to improving patient treatment. Additionally, this module can analyze the data and attributes of a newly registered patient to assign a provisional model until sufficient data are available for building a personalized model. Additionally, this module can be employed to process the data stored in the RAW data database, presenting the information similarly to certain periodic tests conducted on patients at home, such as remote pulse oximetry.

**AI model generation:** The AI model generation module processes data from the RAW data database, which is located in the same cloud, and from the patient’s database. Its responsibility is to generate the patient-personalized AI model capable of predicting patients’ oxygen needs in the near future, thereby deploying a lightweight strategy that may run into a resource-constrained edge device. The resulting AI enables the oxygen dosage to be adjusted dynamically and progressively rather than by relying on a simple closed-loop system. More details on the AI life cycle steps for data preparation, modeling, and deployment are be found in Section 4.

### 3.3. Fog Layer

The fog segment of the architecture is deployed within the reference hospital of each system user. Its main responsibility is that of anonymously linking a patient to the system. The modules comprising the fog layer are described next.

**Professional front end:** The professional front-end interfaces with the functionalities offered by the cloud such as the diagnostic aid module. Through this interface, professionals gain access to information about the patients’ current states and their evolution, as well as comparisons with other patients who are not necessarily from the same hospital, which is all carried out anonymously. This information serves as additional data for analyzing and modifying the patients’ treatments if necessary.

**Patient identity database:** A crucial part that is found in the fog block is that relating to the patient identity database, establishing an anonymous link between the patient and the system. The information required to recognize a patient in the cloud is contained in this database. In essence, this is where the connection with cloud data, the patient’s history, and any other necessary information that could compromise user anonymity are generated. Finally, the patient identity database may either be or not be linked to the user’s medical history database, and different data can be exchanged with it through the professional front end.

### 3.4. Patient Layer

The patient layer, which is the edge of the conceptual system’s architecture, includes all elements closely related to the patient. The two key modules included in this layer will run on a device near the final user that could be a smartphone, an independent microcontroller, or could also be integrated within an oxygen concentrator. A description of each module is presented next.

**Dose prediction:** The dose prediction module uses the connectivity module to download the personalized AI model for the patient from the cloud. Once the model is loaded into the patient device, the module collects data from the patient’s vital signs to be used as input data to generate oxygen titration predictions (as of now, only SpO_2_ predictions), transmitting them to the sensing and connectivity module to be forwarded to the oxygen flow regulator device.

**Sensing and connectivity:** The sensing and connectivity module is responsible for periodically connecting to the cloud, if there is network availability, to check for any new AI models that are available for the patient. If a model update is available, it is downloaded along with the required information for loading, such as input variable scaling or data preprocessing. This module is also in charge of the real-time patient data collection through a Bluetooth-connected pulse oximeter. Upon receiving a patient saturation data point, it sends it to the dose prediction module, and the data are stored in a data block. These blocks are periodically sent to the cloud if connectivity is feasible or are otherwise stored. These data blocks contain many readings with the timestamp of the data collection, patient SpO_2_, heart rate, and the computed saturation prediction. All this information is uploaded to the cloud for future analysis and model training using a unique identifier, which is remarked upon in the subsequent section, to preserve patient anonymity and keep the system’s ease of use.

### 3.5. System Scalability and Privacy

Although many of the key objectives have been previously addressed in the explanation of the architectural layers above, the aspects related to privacy and usability are described here.

Beyond the technical objectives described above, a key objective of the proposed architecture is to be user-friendly for the end user while also aligning to the strong privacy demands required in the health arena. Often, users do not have the knowledge or skills to use technology seamlessly. Therefore, the design is oriented towards ease of use, minimizing user interaction with the system while keeping patient privacy and data security. To achieve this, the fog layer, situated on the hospital premises, will generate a unique identifier (UUID) upon the patient’s registration on the professional front end module, which depends on the edge device’s serial number and the hospital’s private, secret system composition. Later, if needed, this UUID can be regenerated from the hospital side but never from any other part of the system. Upon the generation of this UUID, it is sent to both the patient’s device and the cloud, thus becoming registered in the hospital’s facilities and on the patient’s device. The user identity should be unknown to the cloud and the data will be only kept for the UUID. Consequently, the patient is relieved of the need to remember or type any user credentials and can simply utilize the oxygen concentrator without any other additional complexity.

**Figure 3:**
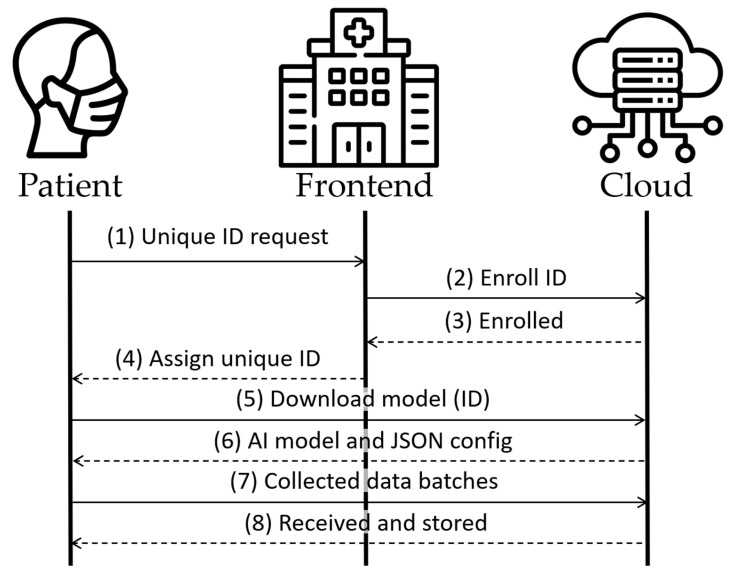
Architecture’s main messages.

Figure 3 illustrates the registration process in the initial four messages. Messages 5 and 6 would recur at hourly intervals to verify the presence of any model update. Finally, messages 7 and 8 show the periodic process, repeating every few minutes, for uploading data collected from the patient to the cloud. All of this occurs if the edge device has network access. Otherwise, the model continues to operate on the edge device, and the data are accumulated in the device until a network connection becomes available. This feature grants the advantage of usability to the system without relying on a continuous network connection, thereby enhancing patient mobility.

Finally, the impact that processing a large amount of data may have on the performance of the architectural concept proposed in this paper is worth mentioning, which is mainly due to the computational burden required. This aspect becomes highly relevant and, consequently, it is included as a key objective to be achieved in the data preparation step of the AI life cycle, as described in the next section.

## 4. AI Life Cycle

Briefly explained, the life cycle of AI is based on three stages as follows: data preparation, AI modeling, and deployment. In the data preparation phase, all available patient data are collected and, based on the parameters deeply described below, various distinct datasets are created, which feature different lengths and prediction horizons. Subsequently, different models can be trained using these datasets and assessed using the fitness function in Equation (2). Once the model that obtained the best results is selected, it is prepared for being deployed for the patient who owns the data used to generate the model.

With the aim of providing a clear and reproducible methodology for AI model generation, the AI life cycle steps are all deeply described.

Firstly, the data obtained from a patient (named as set *R*) from the RAW data database module (see Figure 2) are preprocessed, and *n* datasets {*D*_1_, *D*_2_ …, *D*_n_} are generated encompassing various parameters such as distinct prediction horizons, *f*, different input arrays, *X*, and varying strides, *s*, between data elements. It is worth recalling that, as mentioned at the end of the previous section, one of the objectives of the tests carried out in this paper focuses on reducing the number of datasets required for the proposed workflow to reduce the computational burden needed; therefore, a variety of parameters and the variability of their values are considered. Moreover, with the aim of improving data quality and avoiding inaccuracies from sensor readings, we assume data arrays containing time continuous data, i.e., that of the timestamp difference between two consecutive data points being 3 s at most. The reasoning behind this is that, within a 3 s interval, the data acquired and explored do not show remarkable changes and can be considered as continuous. Nevertheless, if a timestamp difference between consecutive data points is more than 3 s, a new data array is populated.

After determining the appropriate length of the input data size, i.e., the window size, *w_n_*, for the aforementioned continuous data arrays, an intermediate multidimensional matrix is generated and its rows are finally added to the dataset. It is worth noting the resulting dataset consists of the matrix of *w* + 1 columns where *w* represents consecutive data points according to *s*, and an additional column corresponds to the future data point according to *f*. Equation (1) represents the computation of each element, *m_ij_*, of the intermediate matrix given a continuous data array, *X*, and also being *x_i_* with the *i*-th data point in *X*.

(1) $$\begin{array}{c} m_{ij}=\left\{\begin{array}{c} x_{i+s*(j-1)}\;\;\;\;,\;\;\;\;\;\;\;\;j\leq w\\ x_{i+s*\left(j-1\right)+f}\;\;,\;\;\;\;\;\;\;\;j=w+1 \end{array}\right.\\ \begin{array}{c} \forall i\in \left\{1,\ldots ,\left|X\right|-s*\left(w-1\right)-f\right\},j\in \{1,\ldots ,w+1\} \end{array} \end{array}$$

In addition, Algorithm 1 represents the pseudocode for the proposed approach to generate dataset *D* given the set of collected data *R*, window size *w*, stride *s*, and prediction horizon *f*. Moreover, *r* ∈ *R* represents a data point tuple containing, among other things, the SpO_2_ measured value and its timestamp. In brief, after initializing dataset *D* and the continuous data array *X* to *r*_0_, upon the first data point in *R* (line 1), the algorithm either generates the data array *X* with time continuous data points (lines 3–4) or generates a new array by initializing *X* each time two consecutive data points are not considered continuous in time (line 9). Moreover, for each data array *X* that is generated, the corresponding intermediate matrix *M* is computed in *computeIntermediateMatrix*(*·*) according to Equation (1), and its rows are added to dataset *D*, *addRows*(*·*) (lines 6–8 and lines 10–12). It is worth recalling that, in a case where the number of data points in *X* is not enough to cover the window size, *w* (|*X*| < *w*), no rows are added to the dataset from that continuous data array. Finally, the computed dataset is returned (line 13), and the algorithm is executed until the required datasets {*D*_1_, *D*_2_, …, *D*_n_} encompassing various parameters are generated.
**Algorithm 1:****INPUT:**  *R*, *w*, *s*, *f***OUTPUT:**  *D*1:Initialize *D* and *X* ← {*r*_0_}2:**for each** <*r*, *r’*> ∈ *R*
**do**3:       **if** areContinuous(<*r*, *r’*>) **then**4:          *X* ← *X* U {*r’*}5:**else**6:       **if**
*X* ≠ Ø **then**7:          *M* ← computeIntermediateMatrix(*X*, *w*, *s*, *f*)8:          *D* ← addRows(*D*, *M*)9:      *X* ← {*r’*}10:**if**  *X* ≠ Ø **then**11:      *M* ← computeIntermediateMatrix(*X*, *w*, *s*, *f*)12:      *D* ← addRows(*D*, *M*)13:**return**  *D*

In the proposed workflow, a set of AI learning algorithms of *L* = {*l*_1_, *l*_2_, *…*, *l_k_*} is considered (after the preliminary test performed in [25], we can consider fully connected NN and gated recurring unit architectures with and without the heart rate added to the input dataset). Then, a set of AI architectures, *A*, is generated; each architecture has different architectural or hyperparameter configurations. Furthermore, tuples of datasets generated as described in the previous paragraph are characterized by the parameters of *<D*, *w*, *s*, *f>* and are collected into set $\tilde{D}$. With the aim of generating a wide variety of models, all the architectures of each learning algorithm are trained and tested using all the datasets in $\tilde{D}$ (thus resulting in |*A*|·| $\tilde{D}$| models), and the fitness function in Equation (2) is evaluated with the objective of selecting the best architecture and dataset for each learning algorithm.

(2) $$\frac{Max(AE)}{\alpha }+\beta \cdot MSE+\gamma \cdot MAPE+\delta \cdot MAE$$

It is worth mentioning that the detailed study of the optimal selection of values for *α*, *β*, *γ,* and *δ* in Equation (2) is out of the scope of this manuscript.

Algorithm 2 represents the pseudocode proposed to obtain the best model for each patient given the set of learning algorithms, *L*, the set of architectures, *A*, and the set, $\tilde{D}$, containing datasets and their characterizing parameters, i.e., *w*, *s*, and *f*. First, solution, *sol*, is initialized (line 1). Then, for each learning algorithm *l* in *L* and for all architectures based on such an algorithm, subset *A*(*l*), as well as that for all tuples *d* = <*D*, *w*, *s*, *f*> in $\tilde{D}$ (that is, for all datasets), generates the model and evaluates the fitness function defined in Equation (2) (lines 2–7). Note that in this algorithm, *generateModel*(*·*) includes both the training and testing stages to generate the model given the architecture *a* and dataset *d.D*. If the fitness value obtained is the lowest for that learning algorithm, then *best_sol* is updated with that model and dataset (lines 8 and 9). Finally, for each learning algorithm, the best solution obtained, *best_sol*, is added to the solution (line 10) that is returned by the algorithm (line 11).
**Algorithm 2:****INPUT:**  *L*, *A*, $\tilde{D}$**OUTPUT:**  *sol*1:Initialize *sol*2:**for each**  *l* ∈ *L*
**do**3:       Initialize *best_sol*4:       **for each**
*a* ∈ *A*(*l*) **do**5:             **for each**
*d* ∈ $\tilde{D}\;$
**do**6:               *model* ← generateModel(*a*, *d*.*D*) 7:               *value* ← fitnessFunction(*model*, *d*.*D*)8:               **if**
*value* < *best_sol*.*value*
**then**9:                    *best_sol* ← <*value*, *model*, *d*>10:       *sol* ← addToSol(*sol*, *l*, *best_sol*)11:**return**  *sol*

Finally, once the best models for each learning algorithm are obtained, fitness function of each best model is evaluated using the entire respective model’s dataset in those models. The model resulting in the lowest fitness value is then selected as the most suitable model for the given patient and is dispatched to a dedicated application programming interface (API) deployed to serve the mobile application. Alongside the model, a configuration JavaScript Object Notation, JSON, file is created and uploaded to the API, specifying the needed parameters for the model such as data input shape, length, standard deviation, and mean for input scaling. This file serves as a fundamental reference to guide the application on the correct loading and usage of the selected model given that the models can differ significantly among themselves.

## 5. Materials and Methods

According to the preliminary test conducted in [25], we may assess that there is no universally optimal AI model architecture suitable for all patients. Instead, certain architectures outranked others. In this paper, the hypothesis is the infeasibility of creating one generalized dataset shape for an AI prediction model that could obtain good results for all patients. To test this hypothesis with the model’s input data shapes, a second small-scale study is conducted. This test, for the first time, includes the proposed fitness function in Equation (2) to analyze its effectiveness as well as to check its results alongside the errors of the generated models. Finally, to graphically demonstrate the fact analyzed in [25] that a unique model does not fit all patients to predict the SpO_2_, a unique model trained with the data from a single patient was applied to all patients. The next subsections detail the processes of patient data collection in Section 5.1, data processing in Section 5.2, and the AI architecture and training in Section 5.3.

### 5.1. Data Collection

After obtaining clearance from the ethics committee of the Hospital Clínic de Barcelona, a smartphone was provided to five patients with respiratory disorders who were prescribed LTOT and experienced fluctuations in their SpO_2_ levels. Additionally, a pulse oximeter was supplied to these patients. The smartphone was equipped with a specialized app that connects with the pulse oximeter and forwards the acquired data to a secure cloud server. The patients utilized this technology for a duration of 15 days as part of the test. Additionally, a daily exercise plan was tailored to be executed once a day for each patient to diversify the dataset. Patients were instructed to employ the devices during daily activities or exercises while avoiding usage during sedentary activities.

The data were collected using the mobile application that communicates with the pulse oximeter through Bluetooth technology, measuring SpO_2_ and heart rate at 1 Hz. For each data point received, a timestamp was appended to it. The pulse oximeter has a built-in algorithm that checks the readings’ signal quality. If the signal quality is not trusted, the data point collected is discarded, thereby resulting in data gaps that should be processed later on. Once received, the data are stored in data bundles into the app and transmitted to the cloud in chunks for storage, with each individual patient being identified using the pulse oximeter ID.

### 5.2. Data Processing

After the data collection period, five data sets were created for each patient. The datasets were processed, as described in Equation (2), with variations in the number of inputs (window size), strides, and future values (*w*, *s*, *f*) in order to accentuate the differences and to test how these changes impact the model. Firstly, a set of preliminary tests were performed using many different window sizes, strides, and future values to discard the ones that gave the worst results. However, in the analysis presented in this text, we focus on the five configurations that demonstrated superior accuracy across all five patients. These configurations are <*w20*, *s10*, *f20*>, <*w20*, *s10*, *f30*>, <*w20*, *s10*, *f40*>, <*w20*, *s5*, *f20*>, and <*w10*, *s10*, *f20*> where the letter represents the parameter and the number represents its value, e.g., *w20* represents the window size parameter, *w*, of 20 data points.

### 5.3. AI Model Architechture and Training

A fixed test fully connected NN architecture was employed based on our prior experiences in [25], where the input layer was of the same size as the input data pattern (depending on *w*). The output layer comprised a single element representing the predicted saturation at *f* seconds. The hidden layers were 128, 64, and 32 fully connected neurons, respectively, all utilizing the ReLU activation function. The models were trained until halted by a callback function, using MSE to monitor and control the loss function. These models were created 10 times for each data set randomizing the data train, validation, and test partitions in each (70%, 15%, 15%) to ensure that there was no undue variability in the final results.

## 6. Results

This section summarizes and analyzes the most relevant results obtained to evaluate the solution proposed in this paper. First, the different obtained results are presented and, after that, a detailed discussion is provided.

Table 1, Table 2, Table 3, Table 4 and Table 5 summarize the five input datasets obtained for each patient sorted by MAE, named as patient *A–E*. Specifically, these tables show the number of data points in the resulting dataset for the different values of *w* in data points, *s* in elements, and *f* in seconds, errors obtained (MAE, MSE, MAPE), maximum error value obtained in a prediction (Max), and fitness value resulting from the fitness function for the analysis purposes with parameters *α* = 5, *β* = 2.5, *γ* = 1.67, and *δ* = 1.25. The maximum standard deviation obtained in the ten executions for MAE was 0.16.

**Figure 4:**
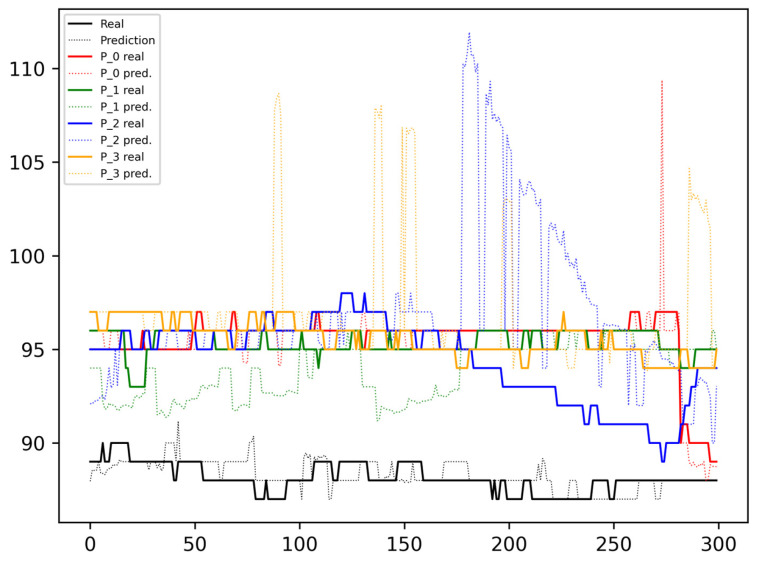
One patient model processing inputs from all patients.

Figure 4 shows the results predicted and measured when a unique model is created based on the data from a single patient, following an input pattern, *w,* of a size of 20 data points, with a stride, *s*, of two elements, and predicting future values, *f*, for 20 s. In the figure, the continuous black line represents the measured (real) values corresponding to the patient owning the model (and data), while the faded line represents the predictions, with *f* shifted to the right to align with the real data. The same visualization scheme with different coloring is considered to represent the measured and predicted values for the other patients.

By analyzing the results shown in the tables above, the dataset obtained with *w* = 20 data points, *s* = 10 elements, and *f* = 20 s achieves lower MAE across all patients, although the differences are not significant. This suggests that this particular dataset configuration could be a suitable option for creating a generic dataset shape applicable to the models. Positive results in three patients are also obtained with *w* = 20 data points, *s* = 10 elements, and *f* = 30 s. No other dataset stands out among the rest.

Examining the amount of data collected from input patterns that have yielded the better and worse results in patients, it can be seen how the result is not closely related with the data volume. Patient B, who achieved the lowest MAE, achieved the best outcome with 52,243 input patterns. In contrast, the patient with the highest MAE obtained the best result from an input containing 45,339 patterns. In short, there was a 15.22% difference in the number of patterns used between them, which may seem substantial. However, when considering Patient D, who obtained the second-best result overall, their optimal result was obtained with 53,802 patterns. This represents a 2.98% increase in data patterns compared with reference Patient B’s data quantity.

As can be seen in Figure 4, the black dotted line, representing the predicted SpO_2_ for the model’s owner, closely aligns with the continuous line depicting the real values. However, errors are more prevalent in data from other patients and are especially high near the peaks and drops. Some moderate deviations can also be observed when changes in the SpO_2_ are not significant.

## 7. Conclusions

Expanding the focus following the obtained results, it can be concluded that these variances are not especially significant, although there are differences among the distinct input datasets. Analyzing a larger sample of patients could potentially identify the input patterns that perform well in a more generalized manner for diverse patients or disease conditions, allowing for their application instead of indiscriminately testing all the possibilities for each patient. This approach would reduce computational load at the expense of a relatively minor increase in errors. Examining the results of Patient A, who exhibits the greatest discrepancy among the top five outcomes with a difference of 0.25 in MAE, this disparity could be nearly nullified by considering the potential error of the pulse oximeter sensor. Consequently, it might be more advantageous to focus on datasets with a longer prediction horizon to provide additional time for the supplied oxygen to travel from the source to the bloodstream.

Allowing a small margin of error, as discussed before, it would also be appropriate to refine the fitness function. Observing the three best results of Patient C, the difference in error is minimal in three different *f* sizes. The optimal result has an f input of 20 s, but the second-best one has an f value of 40 s. Consequently, it would be more beneficial for the patient to use the input pattern with an *f* value of 40 than an *f* value of 20 given the small error differences. This suggests that it would be highly recommended to incorporate a parameter into the fitness function that assigns some weight to the final fitness value.

From the plots in Figure 4, it can be concluded that a model trained with data from one patient cannot be universally applied to another. This does not deny the possibility that among two patients with similar anthropometric characteristics and at a similar stage of their disease, they may share a model at a given moment. However, to analyze and obtain strong conclusions to determine the feasibility of this approach, a larger patient sample is required along with the acquisition of the patients’ specific data that allow for patient clustering.

After the tests with different NN architectures conducted in [25] and the tests presented in this paper, a trend is shown that is considered highly probable for reducing the number of AI model architectures that are tested on each patient, as well as the variety of input data patterns for the model. Consequently, while the most accurate AI model for a patient may not be achieved, the model should be accurate enough. This approach offers the advantage of faster generation and the utilization of fewer resources, thereby providing economic and environmental benefits.

To move further in this paper area, it will be essential to obtain a large number of patients with collected data to draw more robust conclusions. Additionally, acquiring additional data, which are either related to the patient’s disease stage or some other anthropometric attributes, that may facilitate a proper and accurate clustering of patients according to disease stage and similarity may be useful in finding out if a single model could be suitable for a determined cluster of patients while preserving anonymity and adhering to the specific ethical regulations in place.

Moreover, an analysis of which types of neural network architectures are to be developed along with the type of data is crucial for achieving satisfactory results with reduced computational costs. The fitness function should also undergo a thorough review, requiring adjustments and the consideration of incorporating the fitness value to facilitate more efficient selection, thereby balancing the errors and prediction horizon.

As observed in the results section, where the number of data points is not directly linked to the model’s accuracy, it would be beneficial to assess whether some form of data filtering, enrichment, or preprocessing should be applied before direct utilization in the AI model’s training process.

Moving the focus away from the main point of the article, there are still topics to be detailed as the proposed architecture is in its early stages and further exploration is still required in the areas of security, privacy, and applicable regulations in specific communications between components. Additionally, scalability and reliability should be also tested next in the different modules deployed.

Finally, adopting a broader perspective, certain aspects that are outside the scope of this article but deserve attention for further research are considered including, for example, the communication of the oxygen prediction to the oxygen source or the placement of the prediction-generating device and the potential regulations it may need to adhere to. Another observable limitation that was reported by our volunteers during the data collection period is the discomfort felt when wearing the sensor on their finger for extended periods, which is indeed a significant concern. The current wearables like smartwatches do not have a high level of accuracy and are not accepted by the medical community as adequate, accurate, and reliable data collectors for critical health services, as is analyzed in [30]. Nowadays, many research efforts are underway on distinct new technologies [31,32,33] to address this issue and other problems related to SpO_2_ measurements. These novel data collection methods could substantially enhance comfort and foster greater acceptance. These aspects will be progressively defined as our current efforts are oriented towards acquiring data from a higher set of patients in order to achieve the best saturation prediction while also minimizing computational load both in the edge and the cloud.

## Figures and Tables

**Table 1:** Patient *A* results.

*w* Points	*s* el.	*f* sec.	Data	MAE	MSE	MAPE	Max	Fit
20	10	20	45,339	0.89	1.78	0.99	26	12.41
20	10	30	43,617	0.94	2.04	1.05	28	13.64
20	10	40	42,020	0.98	2.22	1.09	28	14.20
20	5	20	71,878	1.02	2.33	1.15	26	14.22
10	10	20	71,878	1.14	2.95	1.29	27	16.34

**Table 2:** Patient *B* results.

*w* Points	*s* el.	*f* sec.	Data	MAE	MSE	MAPE	Max	Fit
20	10	20	52,243	0.49	0.52	0.51	24	7.57
20	10	30	50,687	0.51	0.55	0.53	32	9.30
20	5	20	72,796	0.53	0.65	0.56	27	8.61
20	10	40	49,227	0.53	0.61	0.56	28	8.71
10	10	20	72,796	0.56	0.75	0.59	28	9.14

**Table 3:** Patient *C* results.

*w* Points	*s* el.	*f* sec.	Data	MAE	MSE	MAPE	Max	Fit
20	10	20	83,559	0.61	0.83	0.69	26	9.17
20	10	40	80,992	0.63	0.89	0.71	27	9.59
20	10	30	82,245	0.63	0.90	0.72	27	9.62
20	5	20	99,748	0.63	0.90	0.72	26	9.44
10	10	20	99,748	0.65	0.95	0.74	27	9.80

**Table 4:** Patient *D* results.

*w* Points	*s* el.	*f* sec.	Data	MAE	MSE	MAPE	Max	Fit
20	10	20	53,802	0.85	1.60	0.94	25	11.63
20	10	30	52,187	0.88	1.74	0.96	25	12.05
20	5	20	75,128	0.93	1.92	1.03	26	12.90
20	10	40	50,664	0.94	2.03	1.03	27	13.36
10	10	20	75,128	1.03	2.37	1.15	26	14.32

**Table 5:** Patient *E* results.

*w* Points	*s* el.	*f* sec.	Data	MAE	MSE	MAPE	Max	Fit
20	10	20	101,200	0.52	0.57	0.55	14	5.78
20	5	20	128,041	0.52	0.59	0.56	16	6.26
10	10	20	128,041	0.55	0.67	0.60	25	8.35
10	5	20	147,778	0.56	0.69	0.61	26	8.63
20	10	30	98,965	0.56	0.67	0.61	27	8.79

## Data Availability

Data sharing is not applicable to this article.
